# Tackling Obstacles for Gene Therapy Targeting Neurons: Disrupting Perineural Nets with Hyaluronidase Improves Transduction

**DOI:** 10.1371/journal.pone.0053269

**Published:** 2013-01-03

**Authors:** Klaus Wanisch, Stjepana Kovac, Stephanie Schorge

**Affiliations:** 1 Department of Clinical and Experimental Epilepsy, Institute of Neurology, University College London, London, United Kingdom; 2 Department of Neurology, University of Muenster, Muenster, Germany; University of Sydney, Australia

## Abstract

Gene therapy has been proposed for many diseases in the nervous system. In most cases for successful treatment, therapeutic vectors must be able to transduce mature neurons. However, both *in vivo*, and *in vitro*, where preliminary characterisation of viral particles takes place, transduction of neurons is typically inefficient. One possible explanation is that the extracellular matrix (ECM), forming dense perineural nets (PNNs) around neurons, physically blocks access to the cell surface. We asked whether co-administration of lentiviral vectors with an enzyme that disrupts the ECM could improve transduction efficiency. Using hyaluronidase, an enzyme which degrades hyaluronic acid, a high molecular weight molecule of the ECM with mainly a scaffolding function, we show that *in vitro* in mixed primary cortical cultures, and also *in vivo* in rat cortex, hyaluronidase co-administration increased the percentage of transduced mature, NeuN-positive neurons. Moreover, hyaluronidase was effective at doses that showed no toxicity *in vitro* based on propidium iodide staining of treated cultures. Our data suggest that limited efficacy of neuronal transduction is partly due to PNNs surrounding neurons, and further that co-applying hyaluronidase may benefit applications where efficient transduction of neurons *in vitro* or *in vivo* is required.

## Introduction

In order to understand how different genes can modify specific neuronal functions, it is necessary to manipulate gene expression in neurons. However, reliably introducing genetic material into neurons has been problematic (for review see [1]). Recently lentiviral vectors have emerged as a powerful tool capable of delivering DNA to neurons *in vitro* and *in vivo*. While the transduction efficiency with viral vectors in general is largely dependent on titre, further limitations of the ability to transduce cells are imposed, *in vivo* mainly through the restricted diffusion of lentivector particles around the injection site within the extracellular matrix of the brain [2], and *in vitro,* especially for lower multiplicities of infection (MOIs), through an as yet poorly understood restriction. In many studies this is circumvented by transducing at early time points or simultaneously with seeding cells [3], [4], [5],[6],[7],[8],[9],[10]. In general transduction efficiency is thought to decrease with increasing age of the neuronal culture (days *in vitro*; DIV) but few groups have systematically investigated this. In cases where virally transduced genes are intended to take effect in mature neurons only, or where transduced genes may disrupt development and differentiation of plated neurons, the requirement to transduce neurons *in vitro* at early stages in order to achieve high transduction efficiency presents a major drawback.

One possibility is that the age-related decrease in transduction efficiency is linked to the maturation of neuronal cells, with changes of the outer cellular surface during this phase restricting viral entry into cells. All types of cells are surrounded by extracellular matrix (ECM), and one of the main constituents of the ECM is hyaluronic acid (HA), a long polysaccharide molecule which is composed of N-acetyl-glucosamine and D-glucuronic acid [11], [12]. HA is anchored to extracellular receptor CD44 and CD168 [13] and serves as a scaffold to keep proteins and molecules that support cellular viability in close proximity to the cell surface (for review see [14]). The importance of HA in the brain has been recognised since the 1970s [15], [16]. It entirely covers neurons, including cell bodies, dendrites and axons [17], and in conjunction with other molecules such as chondroitin sulphate proteoglycan and various proteins like tenascins, reelin, laminin, HA is central to building up a net like structure surrounding neurons, known as perineural nets (PNNs). In the brain, HA is thought to maintain the physicochemical properties of the ECM [18], [19], but there is increasing evidence that HA also alters functional properties of neurons [20], [21], [22]: The characteristic distribution of HA and changes during cerebral development are indicative of functional properties (n.b. there is no HA in the adult cerebellum); neurite growth is altered by HA, and neurites tend to avoid HA containing collagen substrates. HA can also have an impact on membrane potential, as indicated by the depolarization observed when HA was added to cultured neurons [23]. The mechanisms of these interactions are still a matter of speculation, but could be related to altered distribution of extracellular ions or signalling via CD44 receptors. Recently HA has been shown to influence neurotransmission and signalling, and also to contribute to synaptic plasticity by regulating use-dependent Ca^2+^ currents via Ca_v_1.2 channels [24], thus manipulation of HA might have consequences for neuronal viability. Hyaluronidase is an enzyme which cleaves HA, and could be used to degrade the PNNs and increase access to the surface of neurons.

We report that treating cells with hyaluronidase improves transduction efficiency with lentiviral vectors *in vitro* and *in vivo*. This was done after evaluating potential toxic effects *in vitro* and *in vivo* on neuronal survival.

## Materials and Methods

All experimental procedures of this study involving animals were carried out in accordance with the UK Animals (Scientific Procedures) Act 1986 and following ethical approval from UCL Institute of Neurology.

Chemicals if not specified are from Sigma (St. Louis, Missouri, USA).

### Production of Lentiviral Vectors and Titration

Second and third generation lentiviral vectors have been produced as described previously [25], [26]. Both express different GFP variants driven by different promoters, with pGIPZ (second generation; Open Biosystems, ThermoScientific, Waltham, Massachusetts, USA) expressing turboGFP (tGFP) under the control of CMV promoter, and pCDH1-MCS1-EF1-copGFP (third generation; System Biosciences, Mountain View, California, USA) expressing copGFP under the EF1a promoter. Packaging was done with plasmids pCMVΔR8.91 for second generation vector [27], or pMDLg/pRRE and pRSV-Rev [26] for third generation vectors, together with the vesicular stomatitis virus protein G envelope plasmid pMD2.G expressing VSV-G surface protein for both (packaging and envelope plasmids were kindly provided by D. Trono, Geneva, Switzerland). To make vectors, the corresponding plasmids were co-transfected with calcium phosphate method into HEK293T cells (originally form ATCC/LGC Standards, Teddington, UK), which were cultured in DMEM (PAA, Pasching, Austria) supplemented with 10% FBS (Gibco-Invitrogen, Carlsbad, California, USA) and Penicillin/Streptomycin (PAA) in a 5%-CO_2_ incubator (Binder, Tuttlingen, Germany) at 37°C in a humidified atmosphere. Supernatant has been collected 48 and 72 hours after transfection, concentrated with ultracentrifugation (Kontron Instruments, Zuerich, Switzerland) in a SW28.1 rotor (Beckman, Brea, California, USA) and resuspended in 50 µl DMEM medium without any additives. Titration was carried out separately for each vector in HEK293T cells by transducing them with serial dilutions of concentrated vector using polybrene (8 µg/ml; Sigma). The percentage of green cells was analysed with a FACS Calibur flow cytometer (Becton Dickinson, Franklin Lakes, New Jersey, USA) 72 h after transduction. The viral dilutions which gave 1–10% green cells have been chosen to calculate titres, which were in the range between 1 * 10^8^ and 2 * 10^9^ TU/ml. As the different vectors relied upon distinct promoters, the calculated titres may partly reflect different reporter expression levels in HEK cells. For this reason, all comparisons of viral efficacy were done using a single virus, however to show that the influence of ECM on transduction level was not restricted to a single promoter we carried out similar tests on more than one vector.

### Rat Primary Mixed Cortical Cultures

Sprague Dawley rat pups (P0–P1; UCL breeding colony, UCL, London, UK) were used for primary mixed cultures. The protocol was a slightly modified version of [28]. Cortex of both hemispheres was removed, purified and minced in HBSS. Trypsin (PAA) was added to a final concentration of 0.25%. After incubation at 37°C for 15 min, cortical pieces were triturated with fire-polished Pasteur pipettes. 10^5^ cells were seeded on 13 mm coverslips (Menzel glass, Braunschweig, Germany), which were pre-treated with Poly D-Lysin/Laminin (Sigma). They were cultivated in 12-well plates in a total volume of 1.5 ml complete Neurobasal-A medium (Neurobasal-A, supplemented with B-27(R) serum-free supplement and Glutamax; all Invitrogen), and kept at 37°C in a humidified atmosphere CO_2_-incubator (5% CO_2_, Binder). A partial medium change (one third of the total volume) to maintain the cultures was scheduled twice per week.

### Hyaluronidase Treatment and Lentiviral Transduction of Rat Primary Neuronal Cultures

Hyaluronidase from bovine testes Type I-S (Sigma) was diluted in complete Neurobasal-A medium as a 3-fold concentrated stock solution, which was done freshly on the day of the experiment. After adding hyaluronidase to the medium it was left on the cells without subsequent medium change (partial medium change was scheduled according to regular maintenance; there were at least two days between experimental treatment and medium change). For transductions, different vector batches were used after correction for differences in their titres. In order to achieve a multiplicity of infection MOI = 1 in a well with 10^5^ cells plated, 10^5^ TU per well were added. Scaling up to higher MOIs was accomplished accordingly.

### Transfection of Neuronal Cultures

For transfection of neuronal cultures in the 12-well format (10^5^ cells per well), Lipofectamine2000™ (Invitrogen) was used according to the manufacturer’s instructions. Two different amounts of DNA were used, with the higher amount being 1.6 µg of pCDH1-MCS1-EF1-copGFP DNA and 4 µl Lipofectamine2000™ reagent in 100 µl OptiMEM® (Invitrogen) each, or the lower amount with 0.8 µg of pCDH1-MCS1-EF1-copGFP DNA and 2 µl Lipofectamine2000™ reagent in 50 µl OptiMEM® each.

### Immunocytochemistry

For immunostaining of transduced neurons *in vitro*, primary neuronal cells grown on a coverslip were fixed in 4% paraformaldehyde/PBS, permeabilized with 0.1% Triton/PBS and subsequently blocked in 1% BSA/0.2% Tween20/PBS. Mouse anti-rat NeuN antibody (1∶400; clone A60, Millipore, Billerica, Massachusetts USA) was used to stain neuron-specific NeuN antigen. Rabbit Anti-turboGFP antibody (1∶1000; AB513, Cambridge Biosciences, Cambridge, UK) was used to stain tGFP and copGFP. Primary antibodies were diluted in 1% BSA/0.2% Tween20/PBS. The corresponding secondary antibodies were Alexa fluor 594™ goat anti-mouse (1∶400, A11005, Invitrogen) and DyLight 488™ goat anti-rabbit (1∶400, 35553, Thermo Scientific), respectively. Cells were mounted on to microscopic slides using Vectashield antifade mounting medium (Vecta Laboratories, Burlingame, California, USA).

### Intracerebral Injections of Lentiviral Vectors and/or Hyaluronidase

Eight male Sprague-Dawley rats (11 weeks; Harlan, Shardlow, UK) were used for these experiments. They were kept in the local animal facilities at the Institute of Neurology, with a 12-h light/dark cycle and with *ad libitum* access to food and water. For stereotactic surgery they were deeply anaesthetised with Vetflurane (Virbac, Suffolk, UK), and fixed to a stereotactic frame (Kopf Instruments, Tujunga, California, USA). Two holes were drilled into the skull at the targeted injection area in the motor cortex. The coordinates were 1.0 mm anterior to Bregma, 2.4 mm laterally from midline, 2.0 mm underneath the surface of the skull. Injections were done with a 33G injection cannula of a 5 µl Hamilton syringe, attached to an automated injection device (WPI, Sarasota, Florida, USA) promoting the injection at a rate of 200 nl/min over a period of 10 min per site. The total injection volume was 2 µl per site. The cannula was left in place for 5 min after each injection. After removal the skin was sutured and disinfected with Braunoderm (Braun-Melsungen, Melsungen, Germany). Postoperative care included analgesia injection of Buprenorphine (Buprenex®, Reckitt Benckiser, Slough, UK). The animals were maintained to allow vector genes 12 days to express prior to brain removal.

The injection solution was a mixture of 1 µl the lentiviral vector (pCDH1-MCS1-EF1-copGFP, titre 2 * 10^9^ TU/ml) and either 1 µl PBS or 1 µl hyaluronidase in PBS (4 or 20 U/µl). This resulted in an injection of 10^6^ TU of lentiviral vector in 2 µl, with or without 4 or 20 U hyaluronidase. Each animal received both treatments, with PBS or hyaluronidase in either of the two hemispheres. Injections of hyaluronidase only contained the final amount of enzyme (4 or 20 U) in 2 µl volume PBS.

### Immunohistochemistry

Animals were transcardially perfused with PBS and 4% PFA in PBS. Brains were removed and fixed overnight in 4% PFA. 50 µm free floating coronal sections were cut on a vibratome (VT1000S, Leica, Wetzlar, Germany) and kept at 4°C in PBS until further use.

Brain slices were permeabilized with 0.3% Triton/PBS, and subsequently blocked in 1% BSA/0.3% Triton/PBS. The same primary (for NeuN and tGFP) and corresponding secondary antibodies were used with the same dilutions as described for Immunocytochemistry. They were diluted in 1% BSA/0.3% Triton/PBS. Stained brain slices were mounted onto microscopic slides and sealed with PVA mounting medium (Sigma).

For staining of the extracellular matrix (see [29]), the slices were incubated in biotinylated *Wisteria floribunda agglutinin* (20 µg/ml; Sigma) in PBS overnight at 4°C. After washing (3×10 min in PBS), slices were incubated in Strepdavidin-Texas Red (5 µg/ml; Invitrogen) for 2 h and washed 3×10 min in PBS afterwards. Nuclear staining was done with 5 µM Hoechst 33342 (Molecular Probes, Eugene, Oregon, USA), slices were mounted onto microscopic slides and sealed with PVA mounting medium (Sigma).

Fluoro-Jade C staining was done as previously described [30]. Briefly, sections were mounted onto microscopic slides and incubated in 0.06% KMnO_4_ for 10 min. After washing (2×2 min in H_2_O), they were incubated in 0.0001% Fluoro-Jade C (in 0.1% acetic acid) for 25 min at RT, washed 3×2 min in H_2_O with the last washing step containing 5 µM Hoechst 33342 (Molecular Probes) for nuclear staining, and finally sealed with DPX mounting medium (Sigma).

### Microscopy and Image Analysis

For the Hoechst/propidium iodide (PI) staining, coverslips with primary cells from rat mixed cortical cultures were incubated in 5 µM PI (Sigma) and 5 µM Hoechst 33342 (Molecular Probes) for 30 min at room temperature. Images were obtained on an epifluorescence inverted microscope equipped with a 20x fluorite objective (Olympus, Tokyo, Japan) using excitation light provided by a Xenon arc lamp. Emitted fluorescence light was reflected through a 380/10 nm filter (for Hoechst) or a 530 nm LP filter (for PI) to a CCD camera (Retiga, QImaging, Canada). Each group was done on 3 coverslips. Each coverslip was analysed on 4 pictures taken in randomly chosen areas using ImageJ software (NIH, Bethesda, Maryland, USA).

Images of lentivirally transduced neuronal cultures were acquired with a standard tissue culture epifluorescence microscope with GFP filter set and 10x or 20x ADL objectives (Nikon) using excitation light provided by an LED excitation light source. GFP-positive cells were counted manually on the entire 13-mm coverslip (for total number of green cells <500) or from the average of 7–10 randomly taken images of the 13-mm coverslip and adjustment of the counted number to the total coverslip size using the microscope’s unique field number (for total number of green cells >500).

Images of double stained neuronal cultures were obtained on an Axiovert AX10 microscope with FITC and Rhodamine filter sets (Zeiss, Oberkochen, Germany), using a 10x Plan-Neofluar objective and excitation provided by a HBO100 mercury light source (Leistungselektronik, Jena, Germany). Overlay analysis was done using Zeiss Axiovision software package.

Images of GFP-only positive brain sections were taken on an Axiovert AX10 microscope with FITC filter set, using a 2.5x Plan-Neofluar objective. Measurements of the size of the injection area were done using ImageJ software. Sections of the entire rostro-caudal length of the injection area were analysed for their two-dimensional spread. The third dimension for the volumetric analysis was added by applying the slice thickness (50 µm) as the distance between sections and calculating the volume accordingly.

Images of double labelled brain slices, slices with staining of the extracellular matrix and slices stained with Fluoro-Jade C were acquired using the confocal laser scanning microscope Zeiss 710 LSM with a META detection system (Zeiss). A 20x objective was used. Hoechst® dye fluorescence was produced with the 405 nm laser, GFP fluorescence using the 488 nm laser, and red fluorescence with the 561 nm laser. For analysis of NeuN/GFP double-positive cells, overlay images of two slices in close proximity to the centre of the injection have been analysed per animal using Volocity Demo software (Perkin Elmer, Waltham, Massachusetts, USA).

### Statistical Analysis

Statistical analysis was done using Origin 8.5 software (Microcal Software Inc., Northampton, Massachusetts, USA). Data are presented as mean ± SEM. Data were compared by Student’s t-test (two-tailed) or analysis of variance (ANOVA), followed by Tukey post-hoc test, if appropriate. Statistical significance was accepted if P<0.05.

## Results

### Transduction Efficiency Decreases with Age of the Culture

**Figure 1:**
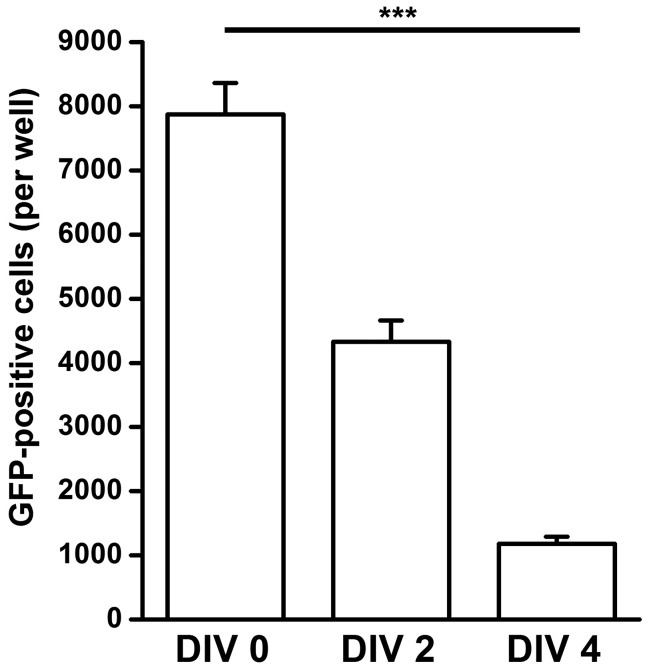
Transduction efficiency decreases with increased age of neuronal culture. Primary neuronal cultures were transduced at DIV 0 - DIV 4 with pGIPZ vector (MOI = 3), revealing decreased transduction efficiency with increasing age of the culture (N = 3 per group). *** P<0.001.

To systematically investigate the relationship between DIV and transduction efficiency, neuronal cultures (10^5^ cells per well; N  = 3 per group) were transduced at different time points after plating, (DIV 0 immediately after plating; DIV 2 and DIV 4, day 2 and day 4 after plating, respectively) with pGIPZ lentiviral vectors at MOI  = 3. The total number of GFP-positive cells was assessed under the microscope seven days after transduction (Fig. 1). The transduction efficiency as observed from the number of green cells, decreased gradually, but significantly from DIV 0 (7872±490) to DIV 4 (1179±109; one-way ANOVA, F_2,6_ = 92.3, P = 0.00003).

### Hyaluronidase has a Dose Dependent Toxicity on Primary Neurons which is Age Dependent

**Figure 2:**
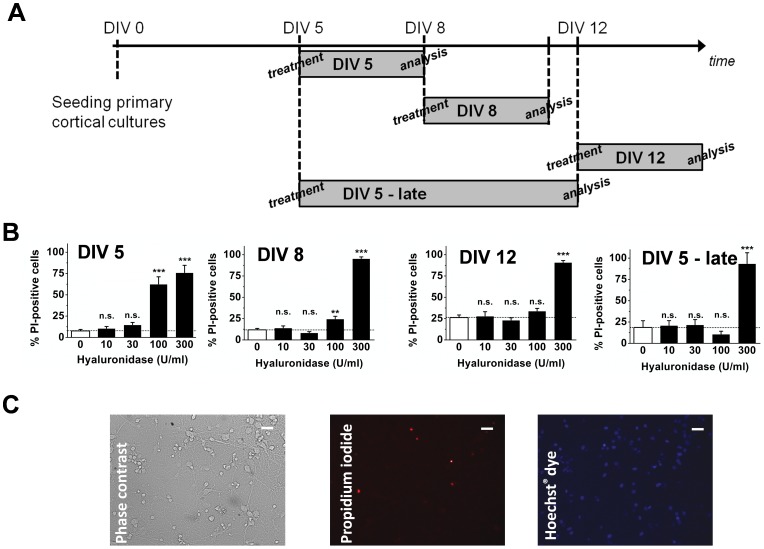
Toxicity of hyaluronidase *in vitro* is dependent on dose and age of neuronal cultures. (**A**) Experimental timeline. For each row, treatment refers to the point at which hyaluronidase was added, and analysis indicates when cells were assessed for toxicity. DIV5– late cells were treated with hyaluronidase at the same time as DIV 5 cells but were allowed to recover for longer. (**B**) Three days after treatment with hyaluronidase, PI/Hoechst staining of neuronal cultures revealed increased cell death at 100 U/ml and 300 U/ml for DIV 5 and DIV 8 cultures, while 100 U/ml did not produce any difference to control level for DIV 12 cells. Allowing cells treated on DIV 5 to recover for 7 days after treatment (DIV 5 - late) showed toxic effects only for 300 U/ml, revealing that potential damage of 100 U/ml is transient. *** p<0.001, ** p<0.01, n.s. not significant, compared to 0 U/ml respectively (ANOVA) (**C**) Representative images of hyaluronidase (10 U/ml) treated cells with PI and Hoechst staining. (N = 3 per group). Scale bar = 10 µm;

There is evidence that HA promotes neuronal survival, which may be reduced by hyaluronidase treatment. To evaluate the toxicity of hyaluronidase treatment neuronal cell cultures of different ages (DIV 5, 8, 12) were incubated with a range of concentrations of hyaluronidase (0 U/ml –300 U/ml) in the growth medium (complete Neurobasal-A). After 3 or 7 days, the viability of cells was determined with PI/DAPI staining (N = 3 per group; Fig. 2A for timeline, 2C for example pictures). The two lower concentrations (10 and 30 U/ml) did not show any difference compared to control conditions (0 U/ml), with the means of % PI-positive cells for 0 U/ml being 7.53±1.76% on DIV 5 (10 U, P = 0.99; 30 U, P = 0.87), 11.74±1.7% on DIV 8 (10 U, P = 0.90; 30 U, P = 0.80) and 26.12±3.11% on DIV 12 (10 U, P = 0.99; 30 U, P = 0.97). The highest concentration (300 U/ml) resulted in high levels of PI-positive cells for all DIVs (76.2–95.6%), while 100 U/ml was toxic for DIV 5 (P = 0.00003) and DIV 8 (P = 0.0029) neuronal cultures with 62.75±8.37% and 24.7±2.95% PI-positive cells respectively. For DIV 12 cultures there was no difference to control (P = 0.45). Two-way ANOVA (DIV, hyaluronidase concentration) analysis, followed by Tukey post-hoc tests confirmed a significant effect of DIV (F_2,45_ = 7.7, P = 0.001), hyaluronidase concentration (F_4,45_ = 191.0, P<10^−8^) as well as the interaction between hyaluronidase concentration and DIV (F_8,45_ = 10.1, P = 6.5*10^−8^). To test for possible side effects of hyaluronidase treatment at a later time point, DIV 5 treated cells were also investigated 8 days after treatment. This revealed essentially the same pattern as found on DIV 12, with 10, 30 and 100 U/ml resulting not in more PI-positive cells than control (P = 0.61; Fig. 2B).

Based on these findings, in the following experiments, the two lower concentrations 10 U/ml and 30 U/ml, which did not produce significant toxic effects, were chosen to investigate effects on transduction.

### Hyaluronidase Improves Transduction Efficiency for Neuronal Cultures DIV 5 - DIV 12

**Figure 3:**
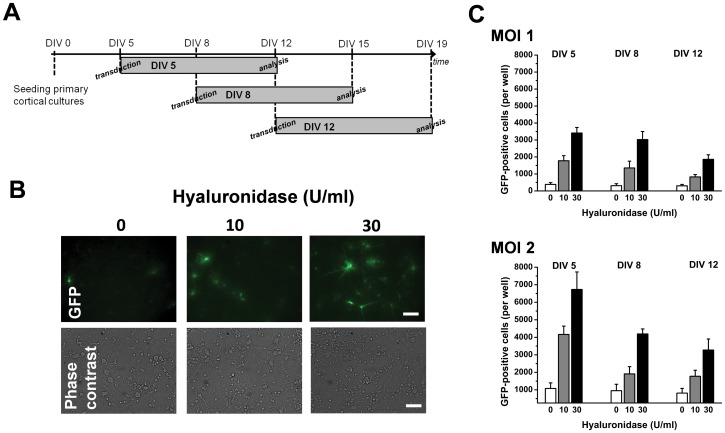
Hyaluronidase increases transduction efficiency of neuronal cultures with higher DIV. (**A**) Experimental timeline as for Figure 2, except that treatment and transduction occurred at the same timepoint (**B**) The two non-toxic doses 10 and 30 U/ml showed robustly improved transduction efficiency on neuronal cultures. (**C**) For the two different MOIs studied, the improved transduction efficiency under Hyaluronidase treatment appeared more pronounced for younger cultures (N = 3 per group; statistics in text). Scale bar = 20 µm.

Transduction of neuronal cultures (10^5^ cells per well; pGIPZ vector) was carried out with different concentrations of hyaluronidase (0, 10 and 30 U/ml) added to the transduction medium (complete Neurobasal-A). The number of green cells was assessed seven days after transduction (Fig. 3A for timeline, Fig. 3B for sample pictures). Detailed analysis revealed (Fig. 3C) for MOI = 1 that there was a significant increase in number of transduced cells which depended on hyaluronidase concentration (F_2,26_ = 57.7, P = 1.5*10^−8^) as well as age of the cultures (DIV, F_2,26_ = 7.4, P = 0.005; two-way ANOVA (DIV, hyaluronidase)). Similar results were obtained using a larger amount of vector (MOI 2), with hyaluronidase concentration increasing (F_2,26_ = 42.2, P = 1.6*10^−7^) and age of culture reducing (F_2,26_ = 13.7, P = 0.0002) efficiency of transduction (N = 3 per group).

### Hyaluronidase Improves Transfection with Lipofectamine2000™

Hyaluronidase may improve transduction efficiency by facilitating the access to the outer cellular surface for the DNA carrier particles. Alternatively, it may have a function which is restricted to lentivirus, for example by increasing the affinity of unique surface properties specific for viral vectors. To rule out a viral specific mechanism, a standard Lipofectamine2000™ transfection of neuronal cultures with pCDH1-MCS1-EF1-copGFP was analysed. The average size of Lipofectamine2000™ particles carrying the DNA are between 160–410 nm diameter [31], and thus slightly larger than the average size of lentiviral vectors (75–100 nm) [32]. However, if hyaluronidase improves access to the cell membrane by increasing the permeability of the ECM, it may be expected to have an effect on other particles even if slightly larger.

**Figure 4:**
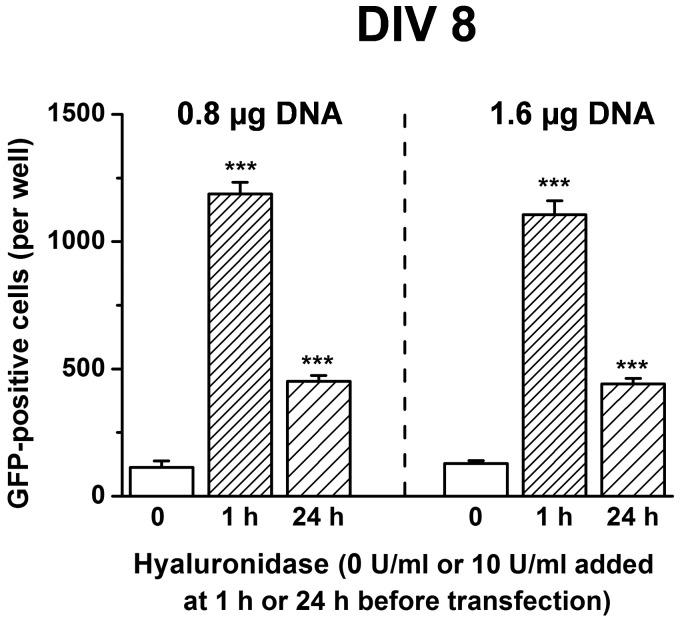
Transfection efficiency of neuronal cultures is increased when preceded with Hyaluronidase treatment. Neuronal cultures were transfected with Lipofectamine2000™ and two different amounts of DNA (0.8 and 1.6 µg DNA), resulting in similar transfection efficiencies, with both transfections strongly enhanced by hyaluronidase treatment. Pretreating cultures 1 h before transfection was more effective than treatment 24 h before transfection. (N = 4 per group) ***p<0.001, compared to 0 U/ml respectively.

Cells were seeded at a density of 10^5^ cells per well, and Lipofectamine2000™ transfection was carried out on DIV 8 (N = 4 per group). The number of green cells was counted 10 days after transfection (Fig. 4). At both low (0.8 µg), and high (1.6 µg) DNA concentrations, treatment with 10 U/ml hyaluronidase significantly increased the efficiency of transfection (1188±45 versus 113±25 green cells per well for 0.8 µg DNA (P<10^−8^) and 1106±54 versus 128±12 green cells per well for 1.6 µg DNA (P<10^−8^). One-way ANOVAs (treatment) confirmed significant differences between treatments for low DNA amount (F_2,11_ = 285.3, P<10^−8^) and high DNA amount (F_2,11_ = 212.8, P = 2.6*10^−8^). Treatment with enzyme for 1 hour was more effective than 24 hour treatment, however 24 hour treatment was still significantly higher than control (451±23 green cells per well for 0.8 µg DNA, P = 1.5*10^−5^, and 442±21 green cells per well for 1.6 µg DNA, P = 4.0*10^−5^; Tukey post-hoc tests).

### Hyaluronidase Increases the Percentage of Transduced NeuN-positive Neurons *in vitro*

**Figure 5:**
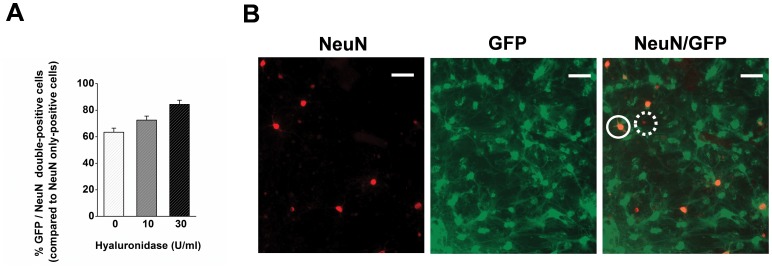
Hyaluronidase increases the percentage of NeuN-positive cells being transduced at high MOI, mimicking an *in vivo* situation with high MOI surrounding the injection site. (**A**) Hyaluronidase increases the percentage of transduced NeuN-positive cells. (**B**) Representative images and overlay. Closed circle represents transduced NeuN-positive cell, dashed circle indicates non-transduced NeuN-positive cell (N = 3 per group). Scale bar = 20 µm.

In order to verify that the improvement in transduction efficiency was not restricted to increase in transduction of non-neuronal cells in our mixed cultures, we also assessed the proportion of transduced cells which were positive for NeuN, a neuronal marker. Primary neuronal cultures were transduced on DIV 12 with the third generation pCDH1-MCS1-EF1-copGFP lentiviral vector at MOI = 30, applying 0, 10 or 30 U/ml hyaluronidase (N = 3 per group), and the number of green cells per well was assessed seven days after transduction (Fig. 5B). This relatively high MOI was chosen in order to produce conditions which are comparable to the situation *in vivo*, where locally high amounts of virus accumulate due to restricted diffusion within the target tissue from the injection needle. For the estimation of an approximate MOI we considered the cerebral cortex to contain around 0.5–1 * 10^5^ neurons per mm^3^ [33], and an injection of 2 * 10^6^ TU of lentiviral vectors (e.g. 2 µl of vector with a titre of 1 * 10^9 ^TU/ml), which is expected to spread approximately 1 mm^3^ around the injection site. Altogether, this would result in an MOI of approximately 20–40 in total for neurons at the injection site. Under these conditions, the transduction efficiency of NeuN-positive cells increased from 63.4±5.3% (control) to 72.5±5.3% (10 U/ml) and 84.5±5.3% (30 U/ml), as confirmed by one-way ANOVA (F_2,8_ = 11.8, P = 0.008; Fig. 5A).

### Toxic Effects Occur *in vivo* after Treatment with High Concentrations of Hyaluronidase

**Figure 6:**
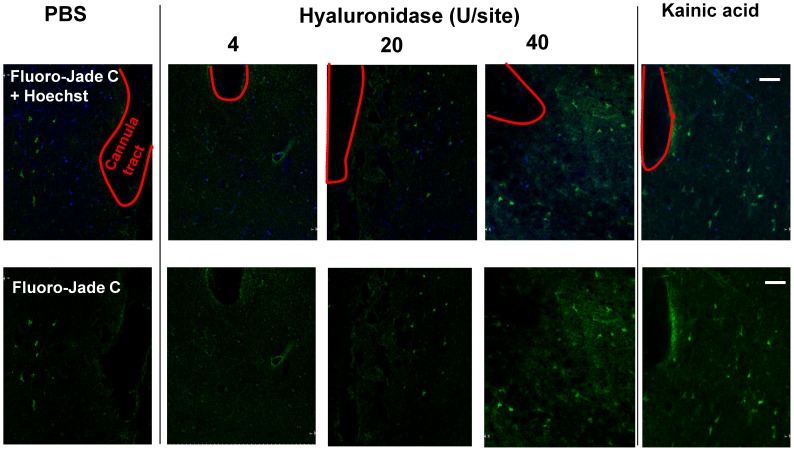
Toxicity of Hyaluronidase *in vivo.* Fluoro-Jade C staining (green, bottom panel) overlaid with Hoechst (blue) reveals differing levels of toxicity after injecting different amounts of hyaluronidase. Compared to PBS control, 4 and 20 U/site showed little or no increase in toxicity for cells around the injection site. In contrast, 40 U hyaluronidase is of a similar extent to kainic acid (4 µg/site), which served as positive control for the staining Pictures were taken from areas surrounding the cannula tract, which is outlined in red. Scale bar = 100 µm.

In order to visualise potentially toxic effects of intracerebral hyaluronidase injections, brains which received PBS, 4, 20, or 40 U hyaluronidase (or 4 µg kainic acid as a positive control) treatment, were perfusion-fixed 24 h after injection, and pictures of the Fluoro-Jade C-stained sections were taken. These images included the injection canal, the area where the hyaluronidase is expected to be at the highest concentration (Fig. 6). In all conditions, a small number of positive cells were visible, including PBS injection and injection of 4 U hyaluronidase. These positive (green) cell bodies were distributed within the area near the canal, but they were also apparent at some distance. In contrast 20 U produced slightly more positive cells, while 40 U resulted in a large number of green cells focused on the area immediately surrounding the injections site. Staining from injections containing 20 U hyaluronidase was similar to the effects of an injection of kainic acid, which is neurotoxic and which served as a positive control.

### Local Injection of Hyaluronidase Produces Local Degradation of Extracellular Matrix

**Figure 7:**
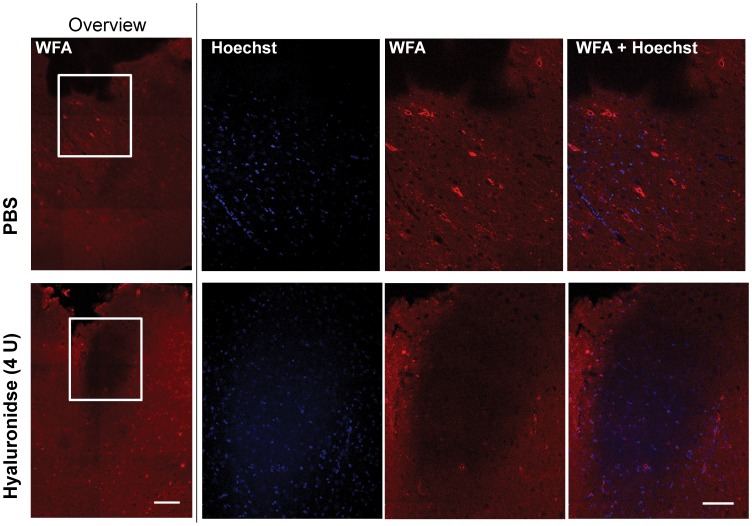
Effects of hyaluronidase on ECM *in vivo.* 24 h after Injection of PBS (top row) or 4 U hyaluronidase (bottom row), ECM staining with WFA/Streptavidin-TexasRed was done. This revealed a distinct area of degraded ECM underneath the cannula tract for 4 U hyaluronidase. Images in leftmost column are lower magnification for overview, scale bar = 250 µm. Right columns are detailed view of the white inserts, scale bar = 100 µm.

For the investigation of the size of the area where the ECM is being degraded, the lower dose of 4 U was chosen because it appears to be less toxic than 20 U with Fluoro-Jade C staining. Brains were analysed 24 h after injection using *Wisteria floribunda agglutinin* (WFA)*/*Streptavidin-TexasRed (WFA), which binds to components of the ECM, and was used to reveal the degradation of ECM, because direct staining of digested hyaluronan in adult brain is not possible [34]. As expected, staining with WFA revealed robust ECM signal across the whole brain section indicated by a red staining between blue (Hoechst® dye) nuclei, and with occasional very high density signals around few selected cells. Injection of 4 U of hyaluronidase produced a clearly visible area lacking ECM signal as a result of the hyaluronidase activity. This area was located slightly below the needle track, consistent with restricted area of hyaluronidase activity. The size of the area affected by hyaluronidase was variable, and the edges were too diffuse to allow exact quantitative analysis, however the largest extent of the diffusion area was in the range of 500–1000 µm in diameter, which would result approximately in a spherical volume of 0.1–0.5 mm^3^ (Fig. 7).

### Intracerebral Injections of Lentiviral Vectors to Assess Effects of Hyaluronidase *in vivo*

**Figure 8:**
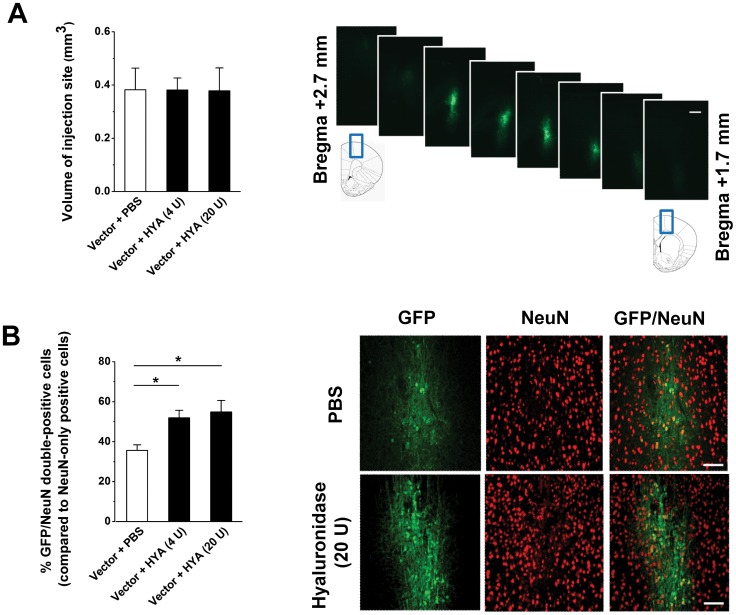
*In vivo* stereotactic injection into rat cortex of pCDH1-MCS1-EF1-copGFP lentiviral vector does not result in wider spreading, but in an increased percentage of transduced NeuN-positive cells. (**A**) Volumetric analysis of the injection site revealed no difference between PBS, 4 or 20 U hyaluronidase (HYA). Inserts with rat brain images were taken from [49]. Scale bar = 400 µm (**B**) Percentage of GFP/NeuN-positive cells is increased after co-injection of viral vector and hyaluronidase. *p<0.05; Scale bar = 100 µm.

A concentrated lentiviral vector stock solution of pCDH1-MCS1-EF1-copGFP (2 * 10^9^ TU/ml) was mixed 1∶1 with PBS or hyaluronidase/PBS (4 or 20 U/µl), and 2 µl of this mixture was injected into the motor cortex of 11-week old rats, resulting in 10^6 ^TU vector and 0, 4 or 20 U hyaluronidase per site. Analysis of the total volume of spread as judged from the GFP-positive area on consecutive brain slices indicated that there was no difference in size of the GFP-positive area with 0.38±0.08 mm^3^ for PBS (N = 7), 0.38±0.05 mm^3^ for 4 U (N = 4) and 0.38±0.09 for 20 U hyaluronidase (N = 3), (P = 0.99; one-way-ANOVA; Fig. 8A). Analysing the proportion of NeuN-positive cells however revealed that there was a significant difference with 54.9±5.7% for 20 U, 51.9±3.7% for 4 U and 35.6±2.8% for PBS of NeuN cells being transduced with lentiviral vector (F_2,11_ = 8.7, P = 0.005; one-way ANOVA; Fig. 8B).

## Discussion

To obtain optimal gene therapy with viral vectors it is important to obtain the best possible transduction efficiency, and to be able to modify the desired subset of cells. In some tissues this presents more of a challenge than in others, and in many circumstances, mature neurons are difficult to transduce. Even before viral gene therapy emerged as a means for delivering genetic material to cells, it was known that chemical agents, in particular polycations like polybrene, DEAE-dextran or poly-1-ornithin could enhance viral infectivity [35], [36]. However, the practical use of these agents is limited due to their toxicity. We show that hyaluronidase treatment also improves the efficiency of viral transduction of neurons with lentiviral vectors both *in vitro* and *in vivo*, and can be effective at low concentrations which are not toxic. Importantly, hyaluronidase treatment increases the percentage of transduced cells positive for NeuN, which is a marker for mature post-mitotic neurons [37]. These cells are often the primary target cells in potential applications of gene therapy in neurosciences and are expected to be a major target of gene therapy in clinical neurology.

Hyaluronidase has already been shown to be effective in improving transduction in other tissues, such as studies using anti-tumour retroviruses in cellular pleura models [38], improvement of activity of oncolytic adenoviruses in a mouse tumour model [39], or increased administration of AAV into skeletal muscle [40]. Previous studies of hyaluronidase in the CNS have shown it can increase the distribution of nanoparticles (54 nm diameter), when the particles are injected into rat brain after hyaluronidase treatment [41]. In that study the size of the target area was increased by about 56%. In contrast, we did not see an increased distribution of the viral particles, which could reflect the smaller size of the nanoparticles compared to lentiviral particles, the different brain region targeted (striatum, as opposed to motor cortical area), the more homogeneous distribution of nanoparticles due to their smooth surface (as opposed to that of lentiviral vector particles, containing proteins), or the higher volume and amount of enzyme used in the nanoparticle study (5 µl of 20000 U/ml, which resulted in a total amount of 100 U injected, as opposed to 4 U in 2 µl per site used in our study). However, our *in vivo* results on toxicity indicate that 40 U produces significant toxicity, which might confound the goal of gene therapy. It is important to note that *in vivo* the distribution volume and local concentration of hyaluronidase are not easy to quantify and there may be a substantial concentration gradient of the injected enzyme between the centre of injection and the diffusion edges.

Abordo-Adesida *et al.* demonstrated that *in vivo* there is a saturation effect for transduction, with the transduction efficiency not changed as the number of particles injected increased from 10^6^ to10^7^ particles per site [42]. One possibility is that disrupting the extracellular matrix with hyaluronidase would overcome this barrier, allowing surplus particles to reach more distant target neurons. However, we did not see an increase in the size of the target area in the presence of hyaluronidase. Instead our data indicated the enzyme increased the proportion of neurons within the targeted area that were positive for GFP, suggesting the enzyme enhanced access to these cells. If the extracellular matrix plays an important role in restricting viral tropism, then mature neurons, with a rather rigid and less permeable extracellular matrix, would be predicted to be more difficult to transduce than cells with less dense ECM. This is consistent with what we saw *in vitro*, where the beneficial effect of hyaluronidase treatment gradually declined from DIV 5 to DIV 12, possibly reflecting the meshwork increasing around neurons and finally make up the PNNs together with other components that are not subject to cleavage by hyaluronidase. Glial cells and neurons have both been implicated in building up ECM material and PNNs [43], [44]. It has been shown that between week 2 and 3 *in vitro* cultures start to build up the ECM which is similar to that of the adult brain and mainly based on HA and chondroitin sulphate proteoglycans, [45]. This time window also coincides with our observation that hyaluronidase treatment is effective at improving transduction efficiency during the second week *in vitro*.

Hyaluronidase also improved transfection efficiency of neurons using a standard transfection protocol using Lipofectamine2000™. This suggests that the improvement in efficiency is not specific to lentiviral particles but may be a direct result of making the cell surface more accessible to particles larger than the pores in the intact ECM. In untreated cells the ECM is described to create a network with pores as small as 56 nm [46]. These pores would effectively exclude, lentiviral particles (70–100 nm diameter [32]) and Lipofectamine2000™ particles (160–410 nm [31]) from reaching the surface of cells. Increased accessibility by degrading the network of ECM may be sufficient to allow larger particles to reach and interact with the cell membrane.

Hyaluronidase could potentially have off-target effects that would limit its use. The hyaluronidase used in this study was isolated from bovine testes and cleaves the 1–4 linkage between N-acetylglucosamine and D-glucuronic acid randomly within the HA macromolecule. It also cleaves analogous molecular bonds within the macromolecules chondroitin, chondroitin-4- and -6-sulfates, and dermatan. However, these molecules form part of the extracellular matrix with primarily scaffolding function and, thus, their cleavage is more likely a synergistic by-phenomenon than a drawback for our application.

In gene therapy trials, it will be important to have a delivery mechanism which does not cause toxicity to the target cells. Our data indicate that hyaluronidase, at the doses we used may have no or limited side effects (as seen *in vitro* from the PI/Hoechst staining for doses 10 and 30 U/ml, Fig. 2; and *in vivo* from Fluoro-Jade C staining for doses 4 and 20 U, Fig. 6), and, does not have long-lasting effects (due to the reversibility of the facilitating effect 24 h after hyaluronidase treatment, Fig. 4). These data suggest at the concentrations we used hyaluronidase is not toxic to neurons. In addition even the toxicity measurement of DIV5 treated cells at the later time point (day 7 after treatment; Fig. 2) is reassuring that no long lasting consequences arise from the one-off treatment.

The unique properties of lentiviral vectors, in particular their large gene packaging capacity and their ability to transduce post-mitotic cells like neurons, make them ideal tools to use for gene therapy in neurosciences, even more in conjunction with the advent of non-integrating lentiviral vectors [47], [48], which have a highly improved safety profile in terms of insertional mutagenesis of the host cell genome. Improving their efficiency for transducing neurons is a major goal, and our data suggest that co-administration of lentiviral vectors with hyaluronidase may be one step towards making gene therapy more effective in the central nervous system.
